# Exposing the error hidden in plain sight: A critique of Calder's (1983) group selectionist seed‐dispersal hypothesis for mistletoe “mimicry” of host plants

**DOI:** 10.1002/ece3.10760

**Published:** 2023-11-23

**Authors:** Kyle E. Harms, David M. Watson, Luis Y. Santiago‐Rosario, Sarah Mathews

**Affiliations:** ^1^ Department of Biological Sciences Louisiana State University Baton Rouge Louisiana USA; ^2^ Gulbali Institute Charles Sturt University, Albury–Wodonga Campus Albury New South Wales Australia; ^3^ Department of Ecology, Evolution, and Behavior University of Minnesota St. Paul Minnesota USA

**Keywords:** adaptation, development, group selection, Loranthaceae, mimicry, mistletoe

## Abstract

Some mistletoe species (Loranthaceae) resemble their host plants to a striking degree. Various mechanisms have been proposed for the developmental origins of novel traits that cause mistletoes to appear similar to their hosts, as well as for the adaptive phenotypic evolution of such traits. Calder (1983) proposed a logically flawed group selectionist seed‐dispersal hypothesis for mistletoes to resemble their hosts. Calder's (1983) hypothesis does not provide a viable potential explanation for mistletoe resemblance to hosts.

## Graphical abstract

Some mistletoe species (Loranthaceae) resemble their hosts to a striking degree. Calder (1983) suggested a logically flawed group selectionist seed‐dispersal hypothesis for this phenomenon. Calder's (1983) hypothesis is not a viable adaptive evolutionary hypothesis.

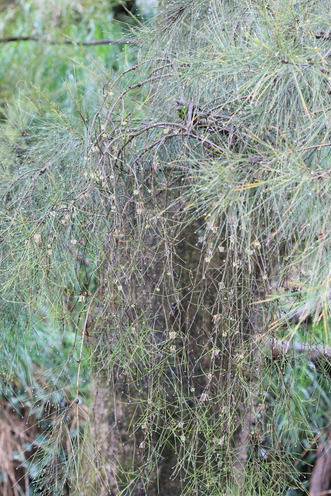

Species within Loranthaceae, a family of hemiparasitic flowering plants, are widely referred to by their common name, “showy mistletoes,” because of their clusters of brightly colored flowers. In contrast to floral showiness, many observers have noted a striking morphological resemblance in vegetative features of some mistletoe species to a particular host species, genus, or broader lineage, which causes those mistletoes on those hosts to be cryptic to human observers (e.g., Moss & Kendall, 2016; Start & Thiele, 2023; Watson, 2019; Figure 1). The similarity between mistletoe and host is so striking in some cases that botanists and ecologists—particularly in Australia, where the phenomenon occurs in multiple species and genera—have proposed several explanatory hypotheses.

**FIGURE 1:**
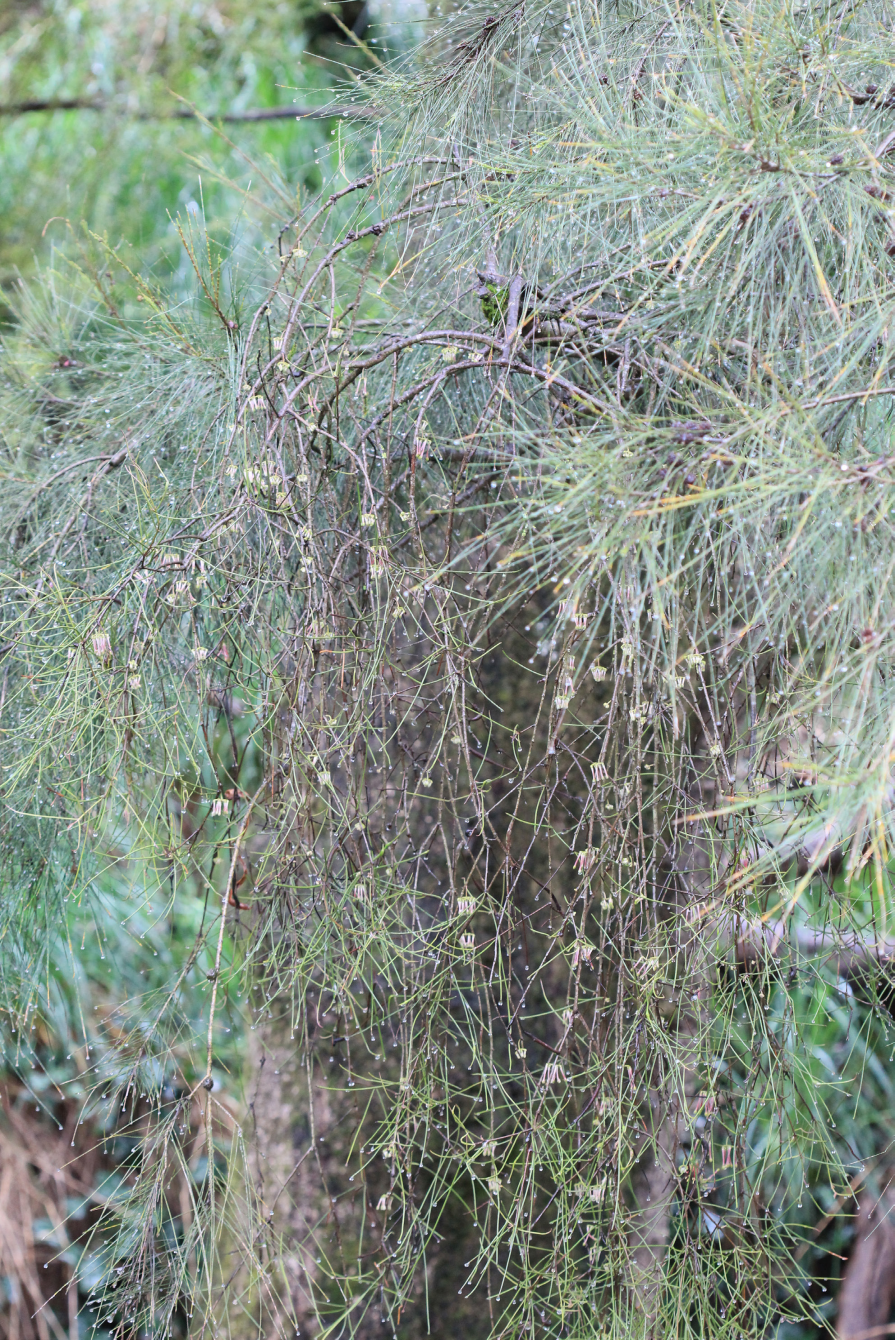
*Amyema cambagei* (Blakely) Danser (Loranthaceae; center) on its host *Casuarina cunninghamiana* Miq. (Casuarinaceae). The mistletoe can be differentiated from its host by its pinkish‐green flower buds. Host foliage is visible across the upper right‐hand corner and partway down the far‐right edge. The photograph was taken on October 8, 2022, at Borenore Karst Conservation Reserve, New South Wales, Australia. © Luis Y. Santiago‐Rosario.

Any given mistletoe species could bear close resemblance to a host species by chance, for example, chance colonization and subsequent spread through a host population by a mistletoe species whose individuals simply happen to appear similar to the host, sans benefit (nor detriment) to the mistletoe because of its similarity to the host. However, Barlow and Wiens (1977) argued that there are too many cases of especially close resemblance in the Australian flora, such that deterministic explanations are more likely than explanations based entirely on chance mistletoe–host pairings.

There are two types of deterministic explanations for a trait or suite of traits that result in a mistletoe's resemblance to a host. First, origins of novel traits are explained by the developmental mechanisms that produce individual organisms (West‐Eberhard, 2003, e.g., p. 201). Second, individuals with specific focal traits can be favored, disfavored, or “ignored” by natural selection, which discriminates among individuals within a population (Darwin, 1859). Accordingly, we organize the various hypotheses for mistletoe resemblance to hosts into two categories: developmental origins of phenotypic novelty and adaptive phenotypic evolution.

## 1 DEVELOPMENTAL ORIGINS OF PHENOTYPIC NOVELTY

A novel phenotype results from novel input during the development of an individual organism, either owing to new genetic material whose trait expression is not otherwise present in the population of conspecifics, or to a change in the environment (West‐Eberhard, 2003). New genetic material could come from mutations, gene flow, or horizontal gene transfer (e.g., non‐sexual movement of genetic information between otherwise separate genomes or gene pools). Went (1971) and Barlow and Wiens (1977) hypothesized genetic transformation (one type of horizontal gene transfer) as a potential mechanism for the origin of novel host‐like traits in mistletoes that resemble their hosts. Environmentally novel inputs to development could include chemical constituents of a mistletoe's host. Atsatt (1979) mentioned “mutation and recombination” as sources of novelty, but rejected those mechanisms in favor of his “host morphogen hypothesis” (Atsatt, 1979, 1983), by which host phytochemicals—such as hormones—induce developmental pathways in mistletoes that are similar to their hosts'. Although horizontal gene transfer is widespread among plant lineages (Bergthorsson et al., 2003), including from parasitic plants to hosts (e.g., Davis et al., 2005) and from hosts to mistletoes (e.g., Skippington et al., 2017), and although hormone induction of phenotypic plasticity occurs in plants (e.g., Voesenek & Blom, 1996), we are not aware of any specific tests of the various hypotheses for the individual‐level developmental origins of host resemblance in mistletoes.

## 2 ADAPTIVE PHENOTYPIC EVOLUTION

Three sub‐categories of hypotheses account for most of the proposed adaptive ideas for mistletoe similarity to hosts: (1) convergence owing to a shared environment; (2) concealment and protection from herbivorous enemies (florivorous, folivorous, granivorous, etc.); (3) improved seed or pollen dispersal by animal vectors.

Evolutionary convergence between a mistletoe species and its host could occur in response to their shared environment (especially the abiotic environment), not because of their physical association with one another. Hemsley (1895–1897) appears to have been the first to suggest this possibility in print (p. 308, “resemblances… due to… local climatal [sic] conditions favorable to the development [presumably evolution] of the same type of foliage”).

Moore (1899) appears to have been the first to suggest in print that evolution within a mistletoe population toward the phenotype of a particular host could result from diminished risks of consumption by herbivores of individuals that are most similar in appearance to their host, that is, concealment and protection from enemies. Although Moore (1899) focused primarily on concealment from florivores, in their review and assessment Barlow and Wiens (1977) favored folivores as the enemies to which concealment could provide a degree of protection.

In contrast to the selective agency of herbivorous enemies, mutualistic seed or pollen vectors could favor mistletoe resemblance to hosts. Barlow and Wiens (1977) suggested that by resembling their hosts, mistletoes could coopt their hosts' seed dispersers or pollinators by virtue of their instinctive attraction to those hosts. Atsatt (1979) proposed that seed dispersers could form search images owing to successful foraging at a mistletoe, which then results in the dispersers seeking to forage in a plant of similar appearance. Mason et al. (2022) provided a framework that helps interpret these seed‐dispersal ideas. Directed dispersal “indicates predictable delivery to favourable microsites,” whereby active directed dispersal occurs when parental plants influence post‐removal propagule fates and passive‐directed dispersal occurs when the plants from which propagules were removed do not (Mason et al., 2022, p. 1908). Accordingly, Barlow and Wiens (1977) hypothesized a mechanism of passive‐directed seed dispersal, whereas Atsatt (1979) hypothesized a mechanism of active‐directed seed dispersal.

We are not aware of any specific tests of the simple convergence hypothesis, nor of the specific seed‐dispersal hypotheses mentioned above. To purposefully evaluate the protective concealment hypothesis, a few comparisons have been made between mistletoe and host leaf‐tissue chemistry and of levels of herbivory among mistletoe and host taxa. For example, Ehleringer, Ullmann, et al. (1986) compared nitrogen levels of 48 mistletoe–host pairs. Cryptic species of mistletoe tended to have higher leaf nitrogen concentrations than their hosts, whereas non‐cryptic mistletoes tended to have less than their hosts, which they interpreted as evidence in favor of the protective concealment hypothesis (Ehleringer, Ullmann, et al., 1986). Atsatt (1979, 1983) surveyed levels of herbivory in the field and assessed foliage preferences of one single individual brushtail possum. Finding low levels of herbivory on mistletoes in the field survey, and a distinct lack of interest in mistletoes by the possum, Atsatt (1979, 1983) rejected the protective concealment hypothesis. Echoing Barlow and Wiens (1977), Canyon & Hill (1997, p. 395) justified their study by the dearth of relevant research on previously proposed hypotheses: “No extended examination of herbivory of host‐parasite pairs has ever been done… to put these explanations to the test.” Canyon and Hill (1997) compared levels of herbivory, leaf nitrogen, water, and toughness, as well as leaf‐shape variability in one cryptic and one non‐cryptic mistletoe and their hosts. Canyon and Hill (1997) claimed that their results “contradict, in some crucial aspect, all of the mimicry hypotheses currently on offer.”

Despite the insights gained from the comparisons mentioned above (plus a few others, for example, Ehleringer, Cook, & Tieszen, 1986; Scalon & Wright, 2015), the data collectively constitute far from conclusive evidence for a general explanation for close resemblance between the many host‐like mistletoe taxa and their hosts. This is especially obvious when we acknowledge that different mechanisms, or combinations of mechanisms, may account for the phenomenon in different lineages. In addition, we are not aware of any phylogenetically informed comparative studies, phylogenetically informed ancestral state reconstructions, nor direct experimental tests of within‐population phenotypic variation (naturally expressed or experimentally produced) of relevant traits combined with their consequences for fitness differences (or their proxies) among individuals, each of which would be especially informative to test the various adaptive hypotheses according to established traditions for doing so (e.g., Endler, 1986; Gould & Lewontin, 1979; Harvey & Purvis, 1991).

## 3 CALDER'S (1983) GROUP SELECTIONIST SEED‐DISPERSAL HYPOTHESIS

Calder (1983) proposed a seed‐dispersal hypothesis for the evolution of host resemblance in mistletoes which differs from the seed‐dispersal mechanisms presented in our earlier summary of hypotheses. His idea is flawed because it is based on group selectionist logic, specifically by requiring individuals throughout the mistletoe population to express the resemblance traits, not necessarily an individual possessor of the traits from which its offsprings' (seeds') fates could confer a fitness advantage to the individual. Calder (1983, p. 14) elaborated his idea as (italics added for emphasis):

Because of the characteristic shape, colour and texture of [host] trees the mistletoe birds will recognize them and search for fruit… *The inter‐tree movements of the mistletoe birds will not be influenced by the availability of mistletoe fruit, because recognition of infections will not be possible at a distance*. Hence the birds will select any [host] tree nearby, infected or not, to search for the fruit of its parasite. This behavior pattern seems well designed to increase the efficiency of specific dispersal to [the host], thus providing an explanation of the evolutionary advantage of cryptic mimicry. To paraphrase: ‘If you need to be dispersed by a fruit‐eating vector to a particular host species then there is great advantage in looking like your host’.

Cook et al. (2020, p. 526) provided additional clarification (italics added for emphasis):

… those mistletoes that most closely resemble their favored hosts would be difficult for their dispersers (predominantly birds) to discern within the canopy. Thus, rather than forming a mistletoe‐specific search image and flying from mistletoe to mistletoe or infected tree to infected tree, *fruit‐eating birds would instead need to search host canopies carefully, prolonging the time spent in the canopy*, and maximizing the probability of seeds from previous meals being deposited.

Calder's (1983) hypothesis is formulated on flawed Darwinian logic. Adaptation by natural selection occurs when individuals bearing a particular trait benefit from their own possession of that trait and pass it along to their offspring through inheritance. Darwin (1859, pp. 5, 61) clearly described the process of natural selection to operate through payoffs to the individuals who bear the traits of interest (italics maintained from the source):

As many more individuals of each species are born than can possibly survive; and as, consequently, there is a frequently recurring struggle for existence, it follows that any being, if it vary however slightly in any manner profitable to itself, under the complex and sometimes varying conditions of life, will have a better chance of surviving, and thus be *naturally selected*. From the strong principle of inheritance, any selected variety will tend to propagate its new and modified form…

Owing to this struggle for life, any variation, however slight and from whatever cause proceeding, if it be in any degree profitable to an individual of any species, in its infinitely complex relations to other organic beings and to external nature, will tend to the preservation of that individual, and will generally be inherited by its offspring. The offspring, also, will thus have a better chance of surviving, for, of the many individuals of any species which are periodically born, but a small number can survive. I have called this principle, by which each slight variation, if useful, is preserved, by the term of Natural Selection…

Note that “useful” in the final quoted sentence specifically refers to the trait's utility to the individual who expresses the trait, not to the population at large, as Williams (1966) and others have explained ever since the Modern Evolutionary Synthesis (Huxley, 1942).

Inconsistent with the Darwinian principle of natural selection, Calder's (1983) logic fails the basic individual‐level test and relies on a group‐selection advantage. For the argument to work under individual‐level selection, traits of an individual mistletoe plant that result in host resemblance would be required to provide that individual with an advantage, but Calder's (1983) idea is that the advantage to a given individual results from *other* individuals within the population expressing the traits. This invites the key question: What would prevent a cheater from being obvious (i.e., not cryptic; see also Atsatt, 1983, p. 264), thereby drawing in dispersers, yet benefitting from the crypsis of others to shape subsequent disperser behavior? Furthermore, a hidden individual mistletoe might go unnoticed by dispersers, thereby reducing its own offsprings' chances of being dispersed in the first place, that is, there could be a net individual‐level seed‐dispersal cost to being cryptic.

Birds dispersing seeds in a population of cryptic mistletoes might spend more time searching for fruit‐bearing individuals and thereby be more likely to deposit any given seed on the branch of a potential host than would be the case for birds searching in a population of non‐cryptic mistletoes. However, the potentially different seed‐dispersal patterns in the two populations would simply be a consequence of the population‐level characteristics of the contrasting populations of mistletoes, not the selective process that would favor crypsis in individual plants. Accordingly, Calder's (1983) seed‐dispersal hypothesis cannot be considered a viable explanation for adaptive phenotypic evolution toward any particular host phenotype within any mistletoe lineage.

## AUTHOR CONTRIBUTIONS

**Kyle E. Harms:** Conceptualization (lead); investigation (lead); writing – original draft (lead). **David M. Watson:** Conceptualization (supporting); investigation (supporting); writing – review and editing (supporting). **Luis Y. Santiago‐Rosario:** Conceptualization (supporting); investigation (supporting); writing – review and editing (supporting). **Sarah Mathews:** Conceptualization (supporting); investigation (supporting); writing – review and editing (supporting).

## Data Availability

This is not applicable to our manuscript, because no data were employed.
